# Commentary: Molecular responses in pig heart to human xenotransplantation unveiled by longitudinal multi‐omic profiling

**DOI:** 10.1002/ctm2.70132

**Published:** 2025-01-08

**Authors:** Brendan J. Keating, Eloi Schmauch, Michael P. Snyder, Jennifer D. Motter, Brian D. Piening

**Affiliations:** ^1^ Department of Surgery NYU Grossman School of Medicine New York New York USA; ^2^ Institute for Systems Genetics NYU Langone Health New York New York USA; ^3^ NYU Langone Transplant Institute NYU Langone Health New York New York USA; ^4^ Department of Genetics Stanford University Palo Alto California USA; ^5^ Earle A. Chiles Research Institute Providence Cancer Center Portland Oregon USA

1

Orthotopic heart transplantation is considered to be the best treatment for end‐stage heart failure, with improved survival and quality of life for patients.^1^ Despite the number of adult and pediatric heart transplants performed in the US having reached >4000 annually, the number of patients waiting for a heart allograft continues to exceed the available supply.^2^ Xenotransplantation has emerged as a promising alternative to address the demand by providing a source of organs that is readily available and practically inexhaustible.^3^ Given their anatomical and physiological similarity to humans, pigs are considered the most suitable donor species for xenotransplantation,^4^ with immunologic barriers steadily being overcome through advances in targeted genetic engineering of the porcine genome and immunosuppression therapies.^4^, ^5^ In 2022, two brain‐dead human recipients “decedents” received 10‐gene‐edited porcine hearts. Over the two ∼3‐day studies, there was sustained cardiac function, without evidence of acute‐onset rejection or zoonotic transmission.^6^ To better elucidate the molecular processes in the peripheral blood and pig heart xenograft tissues, Schmauch and colleagues recently reported in *Nature Medicine* the dynamic molecular interactions following these pig heart to human decedent xenotransplants using comprehensive, longitudinal multi‐omic profiling.^7^ This commentary dissects their key findings, emphasising the clinical translational implications and highlighting future research avenues in this evolving field.

Over the last decade, methodological and technical advances have led to the development of high‐throughput, low‐cost technologies where millions of biomolecules spanning nucleic acids, proteins, lipids and metabolites can be measured simultaneously.^8^ This includes advances in high‐throughput sequencing, with current platforms able to produce large high‐quality human and pig whole genome sequencing (WGS), RNA‐sequencing, epigenetic and genomic structure profiling e.g., bisulfite WGS and Assay for Transposase‐accessible chromatin with sequencing (ATAC‐seq), a technique that assesses chromatin accessibility across the genome. A number of these sequencing approaches can be performed in tissues as well as in cell‐free fractions of DNA and RNA in peripheral fluids including blood and urine. Concomitantly, advances in biochemical preparation and mass spectrometry technologies have enabled the large‐scale quantitative profiling of proteins (proteomics), metabolites (metabolomics) and lipids (lipidomics) in tissues and bodily fluids. Current advances have afforded the ability to perform a variety of assays at single cell resolution, for example, scRNA‐seq and scATAC‐seq, which allow new insights into specific cell‐types and cell–cell interactions that drive different types of rejection which would be otherwise masked at the level of ‘bulk’ RNA profiling. The latest advances in single‐cell analyses also allow spatial cellular profiling in tissues, further adding key contextual information related to donor and recipient cell interactions in vivo.

Leveraging these methodologies, Schmauch and colleagues used peripheral blood mononuclear cells (PBMCs) and pig heart xenograft tissue collected every 6 h to perform an integrated analysis of the transcriptome, lipidome, proteome, and metabolome, as well as histologic and transcriptomic profiling. Their findings revealed distinct molecular signatures. Decedent 1 (D1) exhibited a pronounced immune response characterised by increased CD4+ and CD8+ T cell as well as NK cell activity observed with scRNA‐seq, and upregulation of inflammatory pathways was observed using bulk RNA‐seq. Additionally, an early surge of B cell subtypes, following an increase in plasma cells, was observed, indicating a potential humoral immune response in the early stages of xenotransplantation. In contrast, D2 exhibited a relatively muted response, with a substantial decrease in T cells and stable B cell population after a second dose of rabbit anti‐thymocyte globulin (rATG). Detailed single‐cell transcriptomic analysis of PBMCs revealed a complex interplay of immune cell populations. The increase in CD8+ and CD4+ T cells in D1, along with the pathways, indicates a robust cellular immune response to the xenograft. This observation is further supported by the upregulation of inflammatory pathways in the bulk RNA‐seq datasets. These findings highlight the critical role of immunosuppression in modulating the decedent's immune response and the potential for personalised therapeutic strategies based on individual immune profiles. Thus, in the xenotransplant setting we have confirmatory evidence that immunosuppressant regimes can effectively modulate the immune response. With the advent of multi‐omic technologies ‘druggable’ targets can be identified to help personalise drug development for xenotransplant and/or optimise dosing of immunosuppressant regimens at the pre‐and post‐transplant stages.

These studies also showed ischemia reperfusion injury (IRI) through RNAseq which identified transcriptional signatures, including hypoxia related genes, with spatial transcriptomics showing vascular remodeling. This is important because IRI is a major mechanism by which an inflammatory response can be generated in the heart, which may lead to earlier and more pronounced neoantigen exposure to the immune system, which may amplify immune response(s). Furthermore, this IRI state in the xenograft and the resulting decedent's immune response is an opportunity for intervention e.g. through known interventions/mitigations of IRI such as optimising organ preservation/perfusion to reduce graft dysfunction. Additionally, the integration of single‐nuclei RNA‐seq data from the xenograft with a publicly available dataset of pig hearts subjected to IRI allowed the identification of specific transcriptional signatures associated with IRI in the xenograft. Specifically, the upregulation of hypoxia‐related genes, damage‐associated molecular patterns (DAMP), and inflammatory pathways in D1's heart tissue points to a significant IRI component. Furthermore, spatial transcriptomic analyses revealed a distinct pattern of vascular remodeling and damage in the xenograft, particularly in D1. The enrichment of vascular markers, DAMPs, and inflammatory signals in specific spatial clusters suggests localised tissue injury and repair processes. This observation underscores the importance of optimising organ preservation and surgical techniques to minimise IRI in xenotransplantation. Using additional open‐source RNA profiling from pig heart xenotransplantation in the non‐human primate setting the Schmauch et al. paper showed that the top mRNA and pathway signals were consistent with the phenotype of perioperative cardiac xenograft dysfunction (PCXD), a complication that can occur during a heart xenotransplantation where the xenograft has significant dysfunction immediately after surgery, with expanded T‐cell proliferation evident on the background of IRI.^9^ A size mismatch between the donor heart and D1's chest cavity, coupled with prolonged cold ischemia time and the use of bovine pericardial patches, also exacerbated IRI and contributed to the observed PCXD. PCXD can be managed in the xenotransplant setting using approaches including optimised organ preservation strategies to mitigate PCXD in future xenotransplants.

The importance of this study lies in its use of longitudinal multi‐omics, which provides a powerful framework for elucidating complex molecular events, facilitating a more holistic understanding of biological processes (Figure 1). The findings highlight early molecular and immune responses following xenotransplantation, particularly as they relate to IRI and PCXD. This knowledge can offer critical insights in identifying potential biomarkers and therapeutic targets, including druggable genes, to optimise immunosuppression and patient outcomes. It can also help identify genes that can be knocked out in subsequent porcine models, as well as human transgenes that can be ‘knocked in’ to reduce immune recognition in human patients. Future long‐term studies are needed to assess the longevity and functionality of xenografts, with expansion to other omics layers, such as the epigenome, to further characterise pig‐to‐human heart xenotransplantation. Overall, this study represents a substantial step forward, helping pave the way for successful clinical xenotransplantation.

**FIGURE 1:**
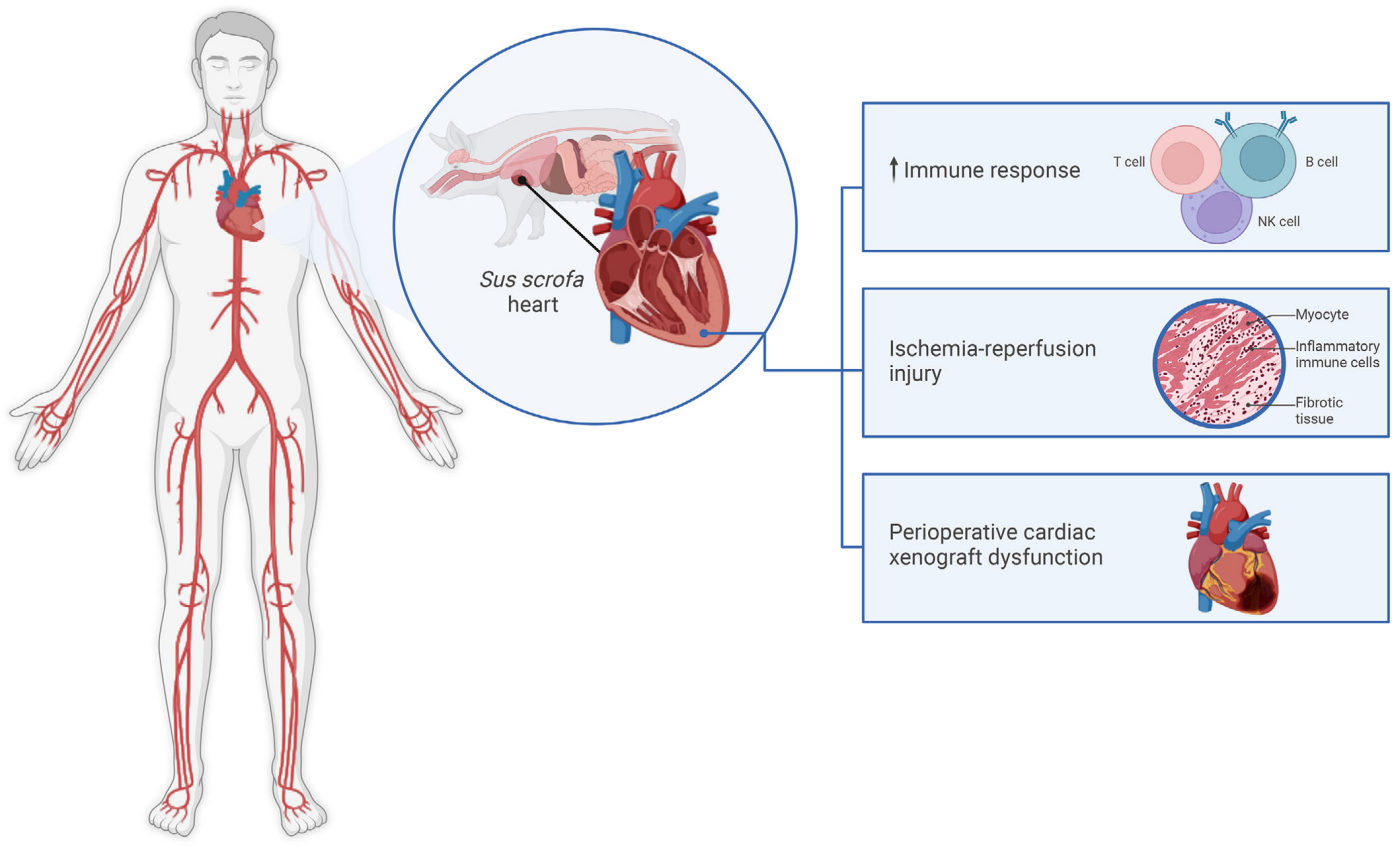
Longitudinal multi‐omic profiling of two porcine hearts transplanted into two brain‐dead human recipients over a 66‐h period: (1) heightened immune response, (2) ischemia‐reperfusion injury, and (3) evidence of perioperative xenograft cardiac dysfunction (Schmauch et al.^7^).

## AUTHOR CONTRIBUTIONS

All coauthors helped with writing and editing of this manuscipt. Michael P. Snyder is a co‐founder and the scientific advisory board member of Personalis, SensOmics, Qbio, January AI, Fodsel, Filtricine, Protos, RTHM, Iollo, Marble Therapeutics, Crosshair Therapeutics, NextThought and Mirvie. He is a scientific advisor of Jupiter, Neuvivo, Swaza, Mitrix, Yuvan, TranscribeGlass, Applied Cognition. The other coauthors have no declarations.
